# 3-hydroxy-3-methylbutyrate (3H3MB) suppresses glioma growth and extends survival in mice

**DOI:** 10.3389/fnmol.2026.1917452

**Published:** 2026-09-17

**Authors:** Rizwan Khan, Xingzi Lin, Francesco Marrocco, Ottavia Giampaoli, Alice Reccagni, Javeria Maqbool, Fabio Sciubba, Adriano Patriarca, Michele De Rosa, Alfredo Miccheli, Gabriele Favaretto, Fabrizio Antonangeli, Cristina Limatola, Giuseppina D’Alessandro

**Affiliations:** 1 Department of Physiology and Pharmacology, Sapienza University, Rome, Italy; 2 Department of Environmental Biology, Sapienza University, Rome, Italy; 3 Nuclear Magnetic Resonance-Based Metabolomics Laboratory, Sapienza University, Rome, Italy; 4 IRCCS, Neuromed, Pozzilli, Italy; 5 Department of Chemistry, Sapienza University, Rome, Italy; 6 Institute of Molecular Biology and Pathology (IBPM), National Research Council (CNR), Rome, Italy; 7 Department of Physiology and Pharmacology, Laboratory Affiliated to Istituto Pasteur Italia, Sapienza University, Rome, Italy

**Keywords:** 3H3MB or β-hydroxy-β-methylbutyrate, glioblastoma, gut microbiota, gut-brain axis, tumor cell invasion, tumor cell proliferation

## Abstract

Glioblastoma (GBM) is the most aggressive primary malignancy of the central nervous system, characterized by poor prognosis, limited therapeutic options, and a high rate of recurrence. A major contributor to its dismal clinical outcome is the highly invasive nature of glioblastoma cells, which infiltrate surrounding healthy brain parenchyma, precluding complete surgical resection and promoting therapeutic resistance. Emerging evidence suggests that the gut microbiota influences glioblastoma progression via modulation of neurotransmitter signaling and immune responses, and through alteration of microbial composition and metabolite production that impact tumor development. Building on our prior work demonstrating the relevance of the gut-brain axis in both physiological and glioma contexts, we identified a significant reduction in fecal levels of 3-hydroxy-3-methylbutyrate (3H3MB), a gut-associated leucine-derived metabolite that is chemically and metabolically distinct from the short-chain fatty acid butyrate, in glioma-bearing mice relative to their pre-tumor state. Given that 3H3MB administration has been reported to attenuate growth and proliferation in carcinoma models, we investigated its potential anti-tumor effects in glioma. *In vivo*, oral gavage of 3H3MB to glioma-bearing mice resulted in a significant reduction in tumor volume, decreased cellular proliferation, and prolonged overall survival compared to control animals. *In vitro*, 3H3MB markedly inhibited the proliferation of mouse glioma and human glioblastoma cell lines, modulating cell cycle phases; moreover, the treatment suppressed glioma cell migration and serum-induced invasion. These findings suggest that 3H3MB exerts robust anti-tumor effects in glioma-bearing mice and holds promise as a novel metabolic therapy for glioblastoma, supported by its established clinical evaluation in other pathological conditions.

## 1 Introduction

Glioblastoma (GBM) is the most common and deadliest primary malignant brain tumor in adults, with patients facing limited treatment options and dismal prognoses (Reni et al., 2017; Aldape et al., 2019). Being characterized by heterogeneous developmental programs, diverse genetic drivers and a complex microenvironment, GBM also exhibits hallmark features such as microvascular proliferation, cellular necrosis, and extensive invasiveness into surrounding parenchyma, contributing to high recurrence rates and mortality among patients (Aldape et al., 2019; McLendon et al., 2008; Tan et al., 2020; Varn et al., 2022). Standard treatment protocols, including surgical resection, radiotherapy, and chemotherapy with temozolomide, are hampered by the infiltrative nature of GBM and the presence of the blood–brain barrier, which restricts the penetration and efficacy of many systemic therapies (Chen et al., 2012; Sadowski et al., 2024; Singh et al., 2004). Emerging evidence suggests that the gut microbiota and its metabolites play significant roles in modulating brain function and influencing cancer progression (Rosito et al., 2024; D’Alessandro et al., 2020, 2021; Dono et al., 2022; Ma et al., 2019).

The gut microbiota has been implicated in the pathogenesis of various intestinal and extraintestinal cancers, including liver, prostate, and lung cancer, through mechanisms involving genetic mutations, immune modulation, and inflammatory signaling (Daisley et al., 2020; Derosa et al., 2022; Ishaq et al., 2024; Yu and Schwabe, 2017). Microbiota-derived metabolites, such as short-chain fatty acids (SCFAs) and amino acid derivatives (e.g., glutamate, glutamine, tryptophan), have been shown to influence brain development, blood–brain barrier integrity, and immune cell dynamics, which may impact tumor growth and progression (Aljarrah et al., 2024; Braniste et al., 2014; D’Alessandro et al., 2020; Wu et al., 2020). Although the relationship between microbiota and gliomas remains largely underexplored, several lines of evidence support the hypothesis that microbiome-derived molecules can either promote or suppress tumor growth (Wang et al., 2024). Experimental studies have demonstrated that gut microbial dysbiosis influences glioma progression, tumor immune surveillance, and the composition of the tumor microenvironment. For instance, depletion or alteration of the gut microbiota affects glioma growth and FoxP3 expression in the tumor microenvironment, while specific intestinal bacteria such as *Bacteroides* have been shown to promote glioma progression by regulating CD8^+^ T-cell infiltration into tumors (D’Alessandro et al., 2020; Fan et al., 2022; Chatterjee et al., 2025). Moreover, transplantation of distinct human gut microbial communities can modify immune cell composition within gliomas and influence the efficacy of immunotherapeutic approaches, including PD-1 blockade (Dees et al., 2021; Green et al., 2025).

Results obtained in animal models revealed biochemical alterations in the feces of glioma-bearing mice, suggesting that glioma induces local and systemic alterations able to modulate gut metabolite profiles (Aljarrah et al., 2024; Dono et al., 2020, 2022). In addition, metabolomic studies have shown that glioma progression is associated with substantial alterations in gut bacterial metabolites, particularly during advanced disease stages, supporting the concept that tumor growth reshapes host-microbiota metabolic interactions (Herbreteau et al., 2022).

Given the emerging evidence that tumor progression can influence host metabolism, we hypothesized that glioma growth may also induce alterations in fecal metabolite profiles. Proton nuclear magnetic resonance (^1^H-NMR) spectroscopy-based metabolomic analysis revealed a different metabolomic profile following glioma growth, with a significant decrease in the concentration of 3-hydroxy 3-methylbutyrate (3H3MB) in the feces of glioma-bearing mice compared to the same mice before tumor induction. Given this reduction, together with previous evidence showing beneficial effects of oral 3H3MB supplementation on brain function *in vivo* (Kougias et al., 2016, 2017), we investigated the functional role of 3H3MB in glioma progression by administering the metabolite *in vivo*. Our results show that oral administration of 3H3MB either before or 1 week after tumor cell inoculation in mice attenuated tumor growth, decreased cell proliferation and extended overall survival. *In vitro*, 3H3MB treatment significantly reduced cell viability in both mouse glioma and human glioblastoma cell lines, modulating cell cycle phases. Additionally, 3H3MB markedly inhibited glioma cell migration and suppressed fetal bovine serum (FBS)-induced invasion in both models. Collectively, these findings demonstrate that 3H3MB can both interfere with early tumor establishment and exert therapeutic activity against established gliomas, supporting its potential as an anti-glioma agent targeting tumor growth, invasion, and progression. Further, these data highlight its therapeutic potential as a novel intervention targeting glioma, particularly considering that 3H3MB has already been extensively evaluated in clinical trials for other pathological conditions, including cancer-induced cachexia, where randomized studies, including phase III trials, have demonstrated its safety and potential benefits in preserving lean body mass and improving clinical outcomes (Berk et al., 2008; Pascoe et al., 2021).

## 2 Materials and methods

### 2.1 Materials

Cell culture medium, including Dulbecco’s Modified Eagle Medium (DMEM), fetal bovine serum (FBS), penicillin G, streptomycin, glutamine, amphotericin B, sodium pyruvate, goat serum, and Hoechst dye, were obtained from GIBCO Invitrogen (Carlsbad, CA, USA). Additional reagents such as 3-(4,5-dimethylthiazol-2-yl)-2,5-diphenyltetrazolium bromide (MTT) salt, dimethyl sulfoxide (DMSO), phosphate-buffered saline (PBS) tablets, Dulbecco’s phosphate-buffered saline, hematoxylin, eosin, and sodium formate were sourced from Sigma-Aldrich (Milan, Italy). The paraformaldehyde solution (4% in PBS) and secondary antibodies were supplied by Santa Cruz Biotechnology. 3H3MB (also known as β-hydroxyisovaleric acid) was purchased by Merck.

### 2.2 Animals and cell lines

All procedures were conducted in accordance with the ARRIVE guidelines (Percie du Sert et al., 2020) and received approval from the local animal welfare body and the Italian Ministry of Health (authorization No 775/2020-PR), adhering to EC Council Directive 2010/63/EU and Italian legislative decree d.lgs.26/2014. Efforts were made to minimize animal suffering and reduce the number of animals used, with sample sizes calculated before experiments commenced. Efforts were maximized to minimize animal distress and to reduce the number of animals utilized per experimental condition. Sample sizes were determined *a priori* to ensure statistical validity while adhering to the principle of reduction. Male 5-week-old C57BL/6N mice were purchased from Charles River (Calco, Italy) and randomly allocated to different experimental groups. Mice were housed two to four per cage and were maintained under a 12-h light/dark cycle in standard cages, with autoclaved bedding and drinking water, and provided with sterilized standard chow *ad libitum*. Continuous monitoring of the microbiological status of both the animals and the facility was maintained throughout the study. The use of male mice only was intended to reduce variability related to female sex hormones. Since estrogen has been reported to influence glioblastoma (GBM) growth (Barone et al., 2009; Sareddy et al., 2012), its effects could impact the reproducibility of the glioma model.

The mouse GL261 glioma cell line was provided by Prof. Michela Matteoli from Humanitas (Milan, Italy). GL261 is a chemically induced C57BL/6 glioma model. The human U87 MG glioma cell line was obtained from the European Collection of Authenticated Cell Cultures (ECACC). SB28-Ohlfest cells were purchased from DSMZ Leibniz Institute, Germany (ACC-880). SB28-Ohlfest was generated from a neonatal female C57BL/6 mouse through Sleeping Beauty-mediated expression of PDGF-B and NRasV12 combined with p53 silencing. CT2a cell line was purchased from Sigma-Aldrich. CT2A derives from a methylcholanthrene-induced astrocytoma in C57BL/6J mice. Human GL15 cells were kindly provided by Dr. E. Castigli, Perugia University, Italy. GL15 cells originate from a glioblastoma of a 59-year-old male patient. Human astrocytes were purchased from ScienCell (HA1800). Cell lines were cultured in DMEM supplemented with 20% (GL261) or 10% heat-inactivated fetal bovine serum (FBS). The culture medium also contained 100 IU/mL penicillin G, 100 μg/mL streptomycin, 2.5 μg/mL amphotericin B, 2 mM glutamine, and 1 mM sodium pyruvate. Cells were maintained at 37 °C in a humidified atmosphere containing 5% CO_2_ and were sub-cultured upon reaching confluency.

### 2.3 Tumor cell inoculation and 3H3MB treatment

Mice were anesthetized with Rompun (20 mg/mL, 75 mg/kg, i.p.) and Zoletil (50/50 mg/mL, 20 mg/kg, i.p.) before being placed in a stereotaxic head frame. Stereotaxic injections of 5.0 × 10^4^ GL261 cells and 5.0 × 10^3^ SB-28 cells were performed. A median incision of approximately 1 cm was made, followed by drilling a burr hole in the skull. The cells were injected into the right striatum (−2 mm lateral and +1 mm anteroposterior from the bregma) at a depth of 3 mm. A total cell suspension of 4 μL in sterile PBS was administered using a Hamilton syringe at a 1 μL/min rate. As previously reported by Nunes et al. (2008), 3H3MB was administered at a dose of 76 mg/kg/day. The treatment began 2 weeks before or 1 week after tumor implantation, 5 days per week until the end of the experiment. 3H3MB was administered in PBS via oral gavage, with each treated mouse receiving 200 μL of the solution. Control mice received 200 μL of PBS alone, administered under the same conditions.

### 2.4 Tumor volume evaluation and immunofluorescence

Twenty-one days post-tumor cell inoculation, glioma-bearing mice were sacrificed, and their brains were isolated, fixed in 4% buffered paraformaldehyde (PFA), and snap-frozen. Coronal brain cryosections, 20 μm in thickness, were prepared using standard procedures and collected at 100 μm intervals. Tumor volume was assessed using hematoxylin-eosin staining, following the manufacturer’s protocol. Briefly, stained brain slices were analyzed using Image Tool 3.0 software (University of Texas Health Science Center, San Antonio, TX, USA). Tumor volume was calculated using the formula (volume = *t* × Σ*A*, where *t* = thickness and *A* = tumor area/slice). Immunofluorescence was performed to quantify proliferating nuclei using the anti-Ki-67 marker. Tissue sections were subjected to antigen retrieval by incubation in boiling citrate buffer for 30 min, followed by a 1 h blocking step at room temperature in 0.5% Triton X-100 and 5% BSA. Sections were then incubated overnight at 4 °C with primary antibodies diluted in blocking solution (Ki-67, clone SP6, Spring Bioscience). After washing, sections were incubated for 1 h at room temperature with an Alexa Fluor 594–conjugated secondary antibody, together with Hoechst for nuclear staining. Finally, sections were mounted using fluorescence mounting medium (DAKO) and analyzed using a Nikon Eclipse fluorescence microscope. The number of Ki-67–positive cells was quantified and normalized to the total number of nuclei for each field of view acquired at 20× magnification using MetaMorph software.

### 2.5 Stool collection and processing

Fresh stool samples were collected from the animals and stored for subsequent analyses. For metabolomic analysis, the samples were rapidly frozen by immersing the tubes containing the stool in liquid nitrogen and then stored at −80 °C until further use.

### 2.6 Fecal water extraction

The still frozen stool samples were added with 1,200 μL of buffer phosphate solution (PBS) prepared in D_2_O and containing NaN_3_ (0.5% v/v) as powerful bacteriostatic substance. Samples were then vortexed in order to achieve a homogeneous solution. After a first centrifugation step at 10,000 × *g* for 25 min at 4 °C, the supernatant was filtered on 40 μm pores filters and added with 200 μL of the same solution of PBS-D_2_O-NaN_3_. A second centrifugation was then performed at same conditions (10,000 × *g* for 25 min at 4 °C) and an amount of 600 μL of supernatant was finally separated for each sample and added to 60 μL of PBS-D_2_O-TSP (2 mM, final TSP concentration) solution, employed as NMR internal standard.

### 2.7 NMR acquirement and processing

All experiments were recorded as previously described (Rosito et al., 2024) at 25 °C on a JEOL ECZR JNM spectrometer equipped with a 14.09 T magnet operating at the proton frequency of 600.13 MHz. ^1^H-NMR spectra were acquired collecting 128 scansions for each sample with 64k data points and a spectral width of 15 ppm. A total of 15 s was set as acquisition time considering a relaxation delay of 7.723 s and guaranteeing complete resonance relaxation between following scansions. A pre-saturation pulse of 2 s has been employed for water signal suppression. Spectra have been processed by applying an exponential window function with a line broadening factor LB = 0.3 Hz. After applying the Fourier transformation, spectra have been manually phased and baseline corrected by applying the BCFR protocol. Metabolites quantitation has been carried out by comparing the integrals of diagnostic resonances with the one of the internal standards (TSP) and normalized by the number of protons according to the general formula:


$$C_{m}=\frac{A_{m}}{A_{I\,S}}\times \frac{H_{I\,S}}{H_{m}}\times C_{I\,S}$$

where C_*m*_ is the concentration of the metabolite, A_*m*_ is the area of the metabolite signal, H_*m*_ is the number of protons generating the metabolite signal, C_*IS*_ is Internal Standard (IS) concentration, A_*IS*_ is the area of the IS signal and H_*IS*_ is the number of protons generating the IS signal. Considering the redundancy of NMR spectra, only those signals free from any overlapping were considered for quantification. All the data were finally expressed as μmol/g of sample.

With the aim of univocally identifying the metabolites in the biological samples, bidimensional experiments such as ^1^H-^1^H Total Correlation Spectroscopy (TOCSY) and ^1^H-^13^C Heteronuclear Single Quantum Correlation (HSQC) and Heteronuclear Multiple Bond Correlation (HMBC) were also performed on selected samples employing the following acquisition parameters. For TOCSY experiments: a spectral width of 9,025 Hz in both dimensions, a data matrix of 8,192 × 256 points, a mixing time of 80 ms, and a relaxation delay of 2 s; for HSQC experiments: a spectral widths of 9,025 KHz and 37,764 KHz for the proton and carbon dimension, respectively, a data matrix of 8,192 × 256 points and a recycle delay of 2 s; for HMBC experiments: a spectral width of 9,025 KHz and 37,764 KHz for the proton and carbon dimension, respectively, with a data matrix of 8 K × 256 points, and a recycle delay of 3 s.

### 2.8 NMR: statistical analysis

Given the high correlation of variables resulting from a metabolomic analysis, the matrix obtained were first considered from a multivariate point of view. To evaluate any samples spontaneous grouping or detect outlier presence unsupervised PCA were applied to the entire dataset after mean-centering and scaling. Besides, to highlight the role of each metabolite in distinguishing between the groups, PLS-DA model have been built, using as validation procedure the Repeated Double Cross Validation (Brown and Forsythe, 1974). Accuracy, Precision, Sensitivity, Specificity and Root Mean Square Error in Cross Validation (RMSECV) were then employed as validation parameters. Finally, only those variables whose regression coefficient show sign coherence during the validation procedure are regarded as significant.

From the univariate analysis point of view, data were primarily tested for normality and homoscedasticity of distributions between classes using Shapiro-Wilk and Brown-Forsythe tests (Stevens, 2007); then *t*-test or Wilcoxon rank sum test were applied accordingly (Bolger et al., 2014). All tests were two-sided, with statistical significance set at *p* < 0.05. All analyzes were performed using in-house written functions running under the MATLAB environment (The MathWorks, Natick, MA, USA).

### 2.9 MTT assay and cell cycle analysis

GL261, U87MG, CT2A, GL-15 cells and human astrocytes were plated in 96-well plates at a density of 5,000 cells per well. SB28 cells were seeded at a lower density (1,500 cells/well). Cells were treated with two concentrations of 3H3MB (100 and 250 μM) to assess cell viability, adding 500 μg/mL MTT salt solution. The resulting formazan crystals were dissolved in DMSO. Optical densities (OD) were then measured at 570 nm (600 nm reference) with a Tecan Infinite F Nano+ plate reader, and the results were processed using Tecan iControl 2.0 software. For cell-cycle analysis, GL261 and SB28 cells were harvested with trypsin-EDTA, transferred into FACS tubes, washed twice with cold PBS−/−, and centrifuged at 1,500 rpm for 5 min. Cells were fixed in 70% ethanol for 1 h at 4 °C and O/N at −20 °C. Cells were incubated with propidium iodide (PI, 50 μg/mL) supplemented with RNase A (40 μg/mL) for 1 h at room temperature in the dark. After the addition of 200 μL PBS−/−, DNA content was measured using a Beckman Coulter CytoFLEX flow cytometer from Beckman Coulter. Data were elaborated using FlowJo software v.10.10.0 (BD) and cell cycle determined using the Dean-Jett-Fox model after doublets exclusion. Cell-cycle distribution was analyzed from DNA content histograms and expressed as the percentage of cells in the G0/G1, S, and G2/M phases.

### 2.10 Wound healing assay

A wound-healing assay was used to assess the migratory capacity of GBM cells under both control and 3H3MB treatment. Equal numbers of cells (5 × 10^4^ cells) were seeded into the inner chambers of cell culture inserts (Ibidi, Germany) placed within a Petri dish. Once the cells adhered to the substrate, the inserts were carefully removed, creating a 500 μm cell-free gap where the cells could migrate. To differentiate between the effects of cell migration and proliferation on wound closure, the cell cycle inhibitor hydroxyurea (5 mM, Sigma-Aldrich) was added during the course of the experiment. Cell migration was monitored by capturing images of the wound areas at specific time points using a CoolSNAP camera (Photometrics) attached to an ECLIPSE Ti-S phase-contrast microscope (Nikon). The images were subsequently analyzed with ImageJ software (NIH). The extent of wound closure was quantified using the following formula: [1 − (empty area at X h/empty area at 0 h)] × 100.

### 2.11 Invasion assay protocol

Fluorescent GL261-RFP and GL15-RFP cells were cultured in Dulbecco’s Modified Eagle Medium (DMEM) supplemented with 10% fetal bovine serum (FBS), 100 IU/mL penicillin G, 100 μg/mL streptomycin, and 25 mM HEPES (pH 7.4). The invasion assay utilized FluoroBlok™ transwell inserts with an 8 μm pore size (Corning^®^) that were coated with the extracellular matrix (ECM) protein diluted in cold, serum-free DMEM. FBS served as the chemoattractant in presence or absence of 3H3MB. Following incubation, the transwell membranes were fixed with 4% paraformaldehyde (PFA), washed with phosphate-buffered saline (PBS), and FluorSave™ reagent was used for mounting membrane face up invading cells on microscope slide with a coverslip. The RFP-labeled cells that had invaded through the membrane were visualized using a fluorescence microscope, and images were captured at a magnification of 20×. Invasion was quantified by counting the number of cells in at least 20 random fields of view per condition.

### 2.12 Statistical analysis

All experiments were performed at least three times. All data are reported as mean ± SEM. GraphPad Prism 8.0 software was used for statistical analysis. Data were analyzed using an unpaired *t*-test when comparing two groups, or one-way ANOVA when comparing more than two groups.

## 3 Results

### 3.1 Glioma-bearing mice have reduced fecal 3-hydroxy-3-methylbutyrate (3H3MB) levels

To investigate whether tumor growth induces changes in fecal metabolites in mice, 1D ^1^H-NMR-based metabolomic multivariate analyzes were first performed in 25 healthy mice. The same animals were subsequently re-analyzed 3 weeks after either glioma cell inoculation (*N* = 15, Glioma) or sham surgery (*N* = 10, Sham), enabling a longitudinal comparison of healthy vs. glioma-bearing and healthy vs. sham-operated mice (Figure 1A and Supplementary Figures 1, 2). Analysis of fecal metabolites through the Venn diagram (Figure 1B), illustrating metabolites that were shared or unique in the multivariate analysis of sham and glioma groups, revealed five metabolites specifically reduced in glioma-bearing mice. This indicates that the observed metabolic profile was associated with glioma growth and was independent of surgical intervention. In particular, the univariate analysis of metabolite abundance revealed that the concentration of 3H3MB was significantly lower in the feces of glioma-bearing mice compared to healthy controls (Figure 1C). These findings suggest that glioma progression is associated with a metabolomic shift involving reduced production or increased utilization of putative gut-associated 3H3MB.

**FIGURE 1:**
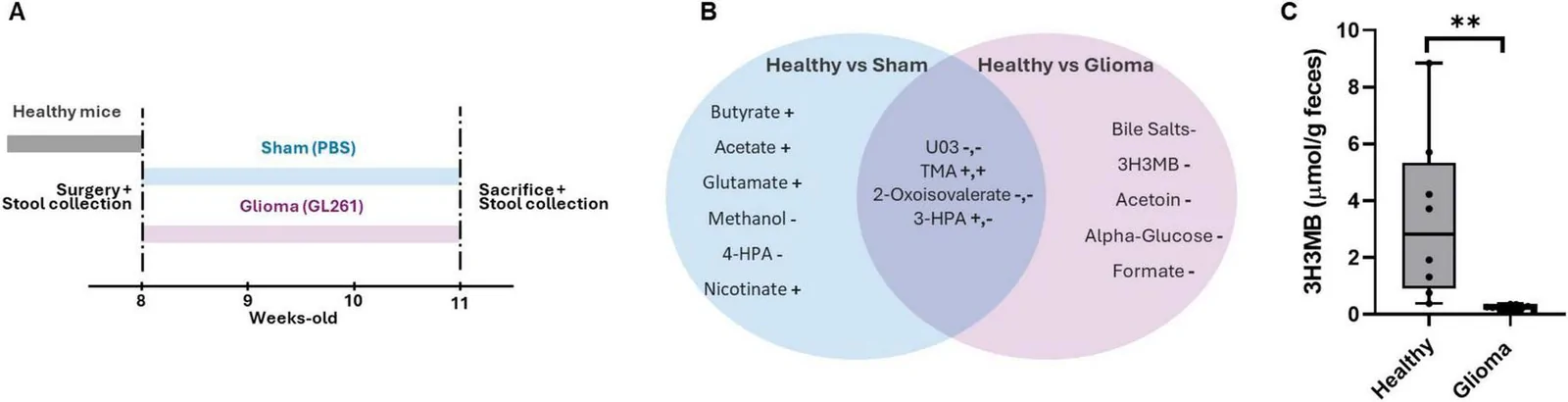
Metabolomic analysis reveals significant alterations in fecal metabolites between healthy and glioma-bearing mice. **(A)** Experimental scheme. **(B)** Venn diagram showing the similarities and differences of metabolites among healthy, sham and glioma mice upon multivariate analysis for the groups (Healthy = 25 divided into Glioma = 15 and Sham = 10 mice). **(C)** Univariate analysis of metabolite abundance (μmol/g feces) in healthy (3H3MB = 3.36 ± 1.01, *n* = 8) and glioma mice (3H3MB = 0.26 ± 0.02, *n* = 9, **p = 0.005 vs. healthy by unpaired Student’s *t*-test). U03, unidentified 03; TMA, trimethylamine; 3-HPA or 4-HPA, 3-hydroxypropionaldehyde.

### 3.2 3 H3MB pre-treatment significantly reduces glioma volume in two mouse models

We investigated the therapeutic potential of 3H3MB pre-treatment using syngeneic mouse glioma models, GL261 and SB-28, implanted into the brain striatum of C57BL/6N mice.

These models differ in immunogenicity and aggressiveness, with SB28 being less immunogenic than GL261 (Genoud et al., 2018). In these mice, 3H3MB was administered via oral gavage (76 mg/kg/day in 200 μL PBS), five times per week over a total period of 5 weeks. Control animals received an equivalent volume of PBS alone. 3H3MB treatment started 2 weeks before tumor cell inoculation and lasted 3 weeks following implantation, as illustrated in Figure 2A. At the end of the 5-week treatment period, mice were sacrificed, and tumor volumes were measured. In the GL261 model, 3H3MB pre-treatment led to a significant reduction in tumor volume (29.32 ± 3.90 mm^3^, *n* = 10) compared to the control group, as shown in Figure 2B (45.20 ± 4.19 mm^3^, *n* = 14; *p* = 0.0135, unpaired Student’s *t*-test). Similarly, in the SB-28 model, 3H3MB-treated mice exhibited significantly smaller tumor volumes (21.00 ± 3.91 mm^3^, *n* = 10) compared to controls (38.20 ± 4.37 mm^3^, *n* = 13; *p* = 0.0110, unpaired Student’s *t*-test), shown in Figure 2C. These findings demonstrate that 3H3MB pre-treatment confers an anti-tumor effect in two different mouse models of glioma, highlighting its ability to interfere with early tumor establishment in GBM progression or recurrence.

**FIGURE 2:**
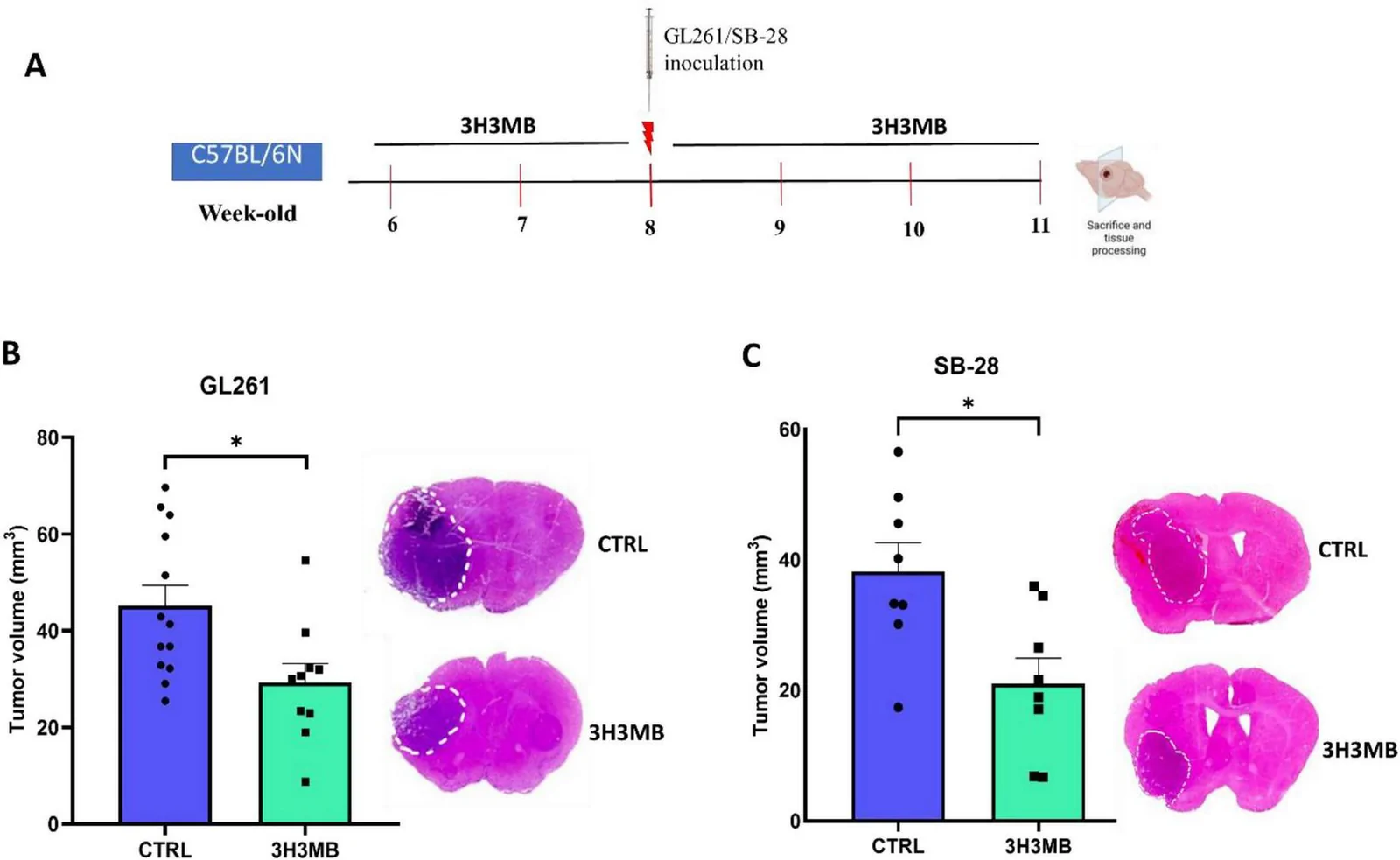
3H3MB oral gavage significantly reduces glioma volume. **(A)** Experimental scheme. C57BL/6N mice implanted with GL261 or SB-28 glioma cells were treated with 3H3MB five times a week for 5 weeks. Treatment started 2 weeks before tumor implantation and continued for 3 weeks. Control mice received PBS alone. **(B)** Tumor volume evaluation in GL261 glioma-bearing mice, treated with 3H3MB (*n* = 10) or vehicle *n* = 14. **(C)** Tumor volume evaluation in SB-28 glioma-bearing mice, treated with 3H3MB (*n* = 10) or vehicle *n* = 13. A unpaired *t*-test was used for statistical analyzes. On the right, representative hematoxylin and eosin stained coronal brain slices illustrate the reduction in tumor size in 3H3MB-treated mice and controls. Data are shown as mean ± SEM. *P < 0.05 by unpaired Student’s *t*-test.

### 3.3 Therapeutic 3H3MB treatment reduces tumor size and proliferation prolonging survival in two glioma-bearing mouse models

To test whether 3H3MB also has therapeutic efficacy after tumor onset, we initiated treatment 1-week post-glioma cell inoculation in the two syngeneic models described above (Figure 3A). Oral 3H3MB significantly reduced tumor volume in both models relative to vehicle controls (GL261, 3H3MB = 7.27 ± 0.16 mm^3^, *n* = 6; CTRL = 11.90 ± 1.67 mm^3^, *n* = 6 *p = 0.0202, unpaired Student’s *t*-test and SB-28, 3H3MB = 36.18 ± 3.77 mm^3^, *n* = 7; CTRL = 68.52 ± 6.19 mm^3^, *n* = 7 ***p = 0.0008, unpaired Student’s *t*-test) (Figures 3B, D). Immunofluorescence analysis of Ki67 expression revealed a marked reduction in proliferating tumor cells in 3H3MB-treated brains (Figures 3C, E), confirming decreased mitotic activity. These results indicate that 3H3MB not only affects the early establishment of the tumor but is also therapeutically effective, attenuating glioma growth and cellular proliferation when administered after tumor establishment. To assess the translational significance of 3H3MB treatment, we conducted survival studies in GL261 and SB28 glioma-bearing mice. Animals received oral 3H3MB (76 mg/kg/day) or vehicle starting 1 week after tumor cell inoculation. Kaplan–Meier survival analysis revealed a significant extension of median survival in both the GL261 (33 days for 3H3MB vs. 27 days for controls; *p* = 0.0040, log-rank test; Figure 3F) and SB28 model of the disease (22 days for 3H3MB vs. 20 days for controls; *p* = 0.0228, log-rank test; Figure 3G). These data demonstrate that oral administration of 3H3MB improves overall survival in preclinical glioma models, supporting its translational potential as an adjuvant therapy for glioblastoma.

**FIGURE 3:**
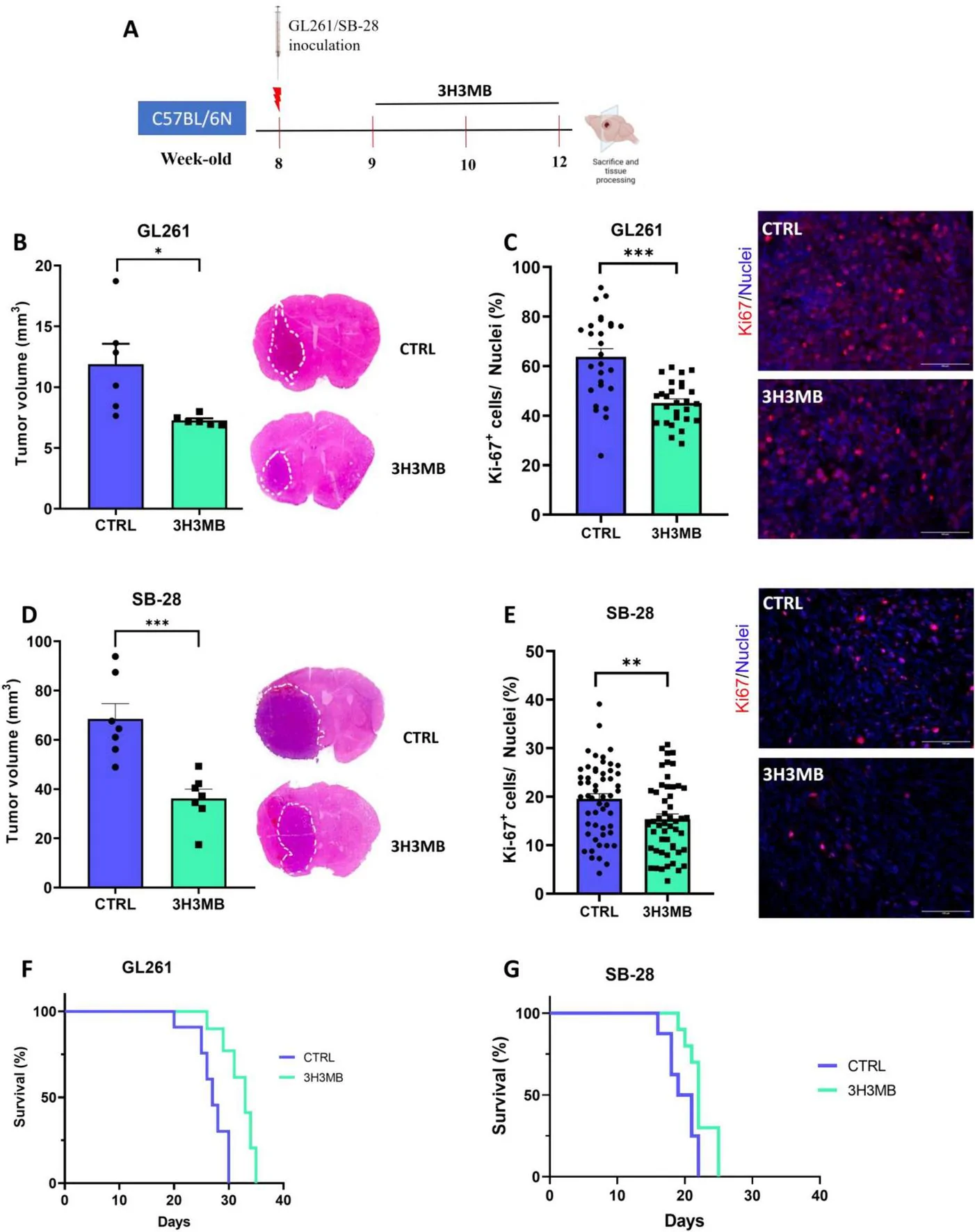
3H3MB oral gavage reduces glioma volume and tumor cell proliferation, increasing survival. **(A)** Schematic of the experimental design: C57BL/6N mice implanted with glioma cells were treated with 3H3MB (76 mg/kg/day in 200 μL of PBS) via oral gavage, starting 1 week after tumor implantation. Treatment continued daily for 2 weeks, while control mice received PBS. Tumor volumes after 2 weeks of 3H3MB treatment in GL261 mice [*N* = 6 per group, *P = 0.0202 by unpaired *t*-test, **(B)**]. Tumor volumes after 3 weeks of 3H3MB treatment in SB-28 mice (*N* = 7 per group, ***P = 0.0008) by unpaired *t*-test, (**D**). Data are shown as mean ± SEM. On the right: representative images of respective H&E-stained coronal brain slices from 3H3MB- or vehicle treated group. **(C,E)** Immunofluorescence analysis of Ki67 showed reduced tumor cell proliferation in 3H3MB-treated brains. On the right: representative images of Ki67 (red) and nuclei (blue) signals in vehicle- or 3H3MB treated group. Kaplan–Meier survival curves of C57BL/6N mice bearing **(F)** GL261 (*N* = 6 per group) or **(G)** SB28 (*N* = 8 controls, *N* = 10 3H3MB) gliomas. A significant improvement in median survival was observed in the 3H3MB-treated groups compared to controls [**(F)**, **P = 0.0040, **(G)**, *P = 0.0228 by log-rank test].

### 3.4 3 H3MB treatment reduces cell viability and modulates cell-cycle progression of glioma cells

The potential direct antitumor effect of 3H3MB was assessed on multiple mouse glioma (GL261, CT2a, and SB-28) and human glioblastoma (U-87 MG, GL-15) cell lines. Cells were treated with two concentrations of 3H3MB (100 μM and 250 μM) for up to 96 h, and viability was quantified using MTT assays. 3H3MB treatment induced a reduction in cell viability across all lines tested. Significant decreases were observed as early as 24 h in human GBM cells (Figures 4D, E) and at 48 h in mouse models (Figures 4A–C; *p* < 0.05–*p* < 0.0001, ANOVA with Tukey’s *post hoc* test). Interestingly, the treatments did not affect normal astrocytes, suggesting that treatment preferentially affects tumor cells (Figure 4F). These findings indicate that 3H3MB exerts consistent antiproliferative effects on gliomas across both human and mouse glioma cells, suggesting a common vulnerability to this metabolite. To determine whether the reduction in cell viability reflected altered proliferative activity, cell-cycle distribution was analyzed by flow cytometry in mouse glioma cells used to create the *in vivo* model of the disease. Treatment with 3H3MB induced a redistribution of cells across the different phases of the cell cycle in both GL261 and SB-28 cells (Figure 5). In particular, both cell lines exhibited a decrease in the G0/G1 fraction associated with an accumulation of cells in the S and G2/M phases, although the magnitude of these changes differed between the two models. These findings indicate that 3H3MB limits glioma cell proliferation by interfering with cell-cycle progression.

**FIGURE 4:**
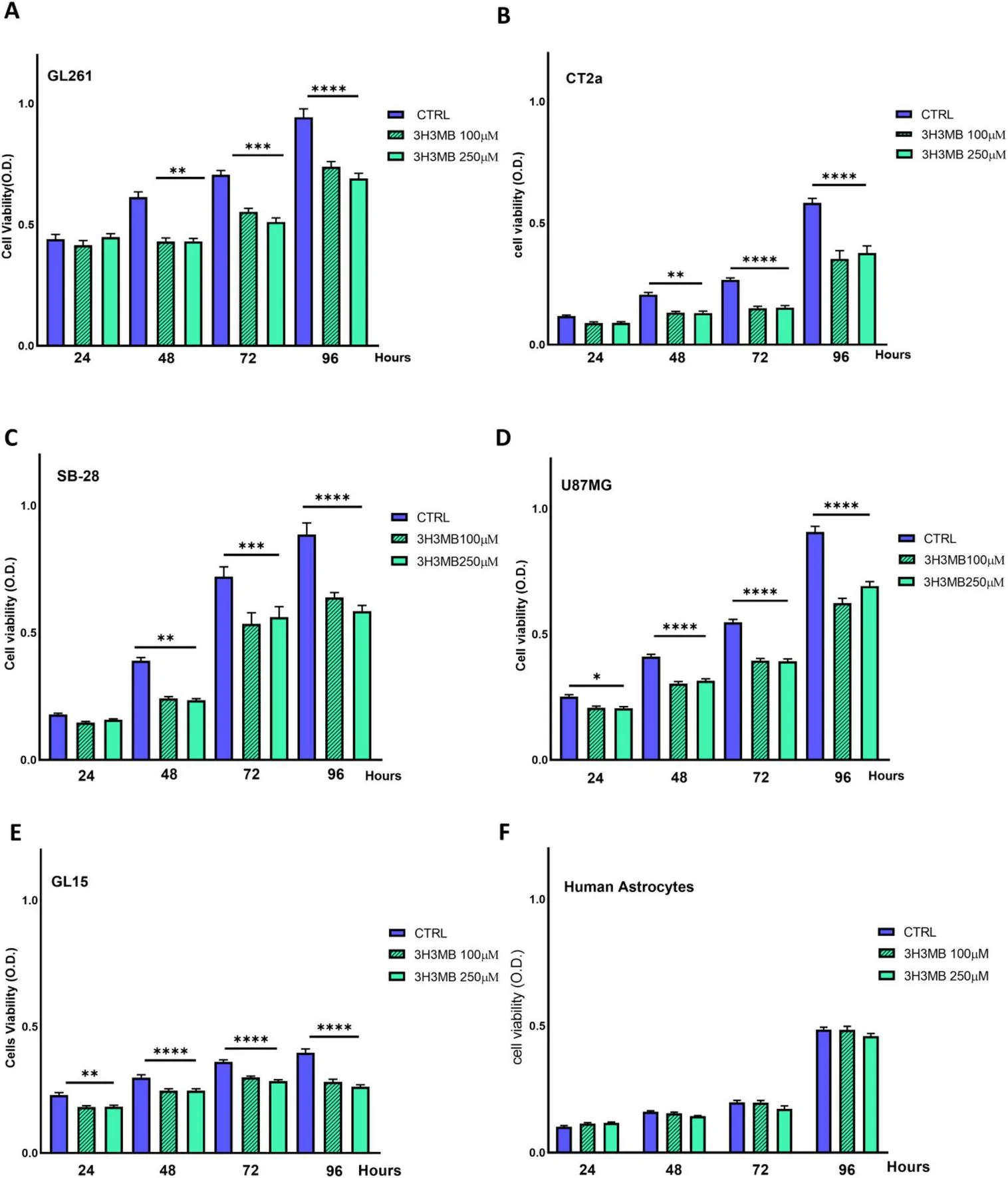
3H3MB treatment reduces cell viability in mouse glioma and human glioblastoma cells. **(A)** GL261, **(B)** CT2a, and **(C)** SB-28 mouse glioma cells, as well as **(D)** U-87MG, **(E)** GL15 human GBM cells and human astrocytes **(F)** were exposed to 3H3MB (100–250 μM) over different time points (24, 48, 72, and 96 h), and cell viability was assessed using the MTT assay. Bar graphs are generated by pooling the data from 3 independent experiments. Error bars represent means ± SEM. Significant reductions in cell viability were observed: *p < 0.05 and ***p < 0.001 in GL261 and CT2a cells; ****p < 0.0001 in U-87MG, GL15, and SB28 cells vs. control. **p < 0.01 vs. control.

**FIGURE 5:**
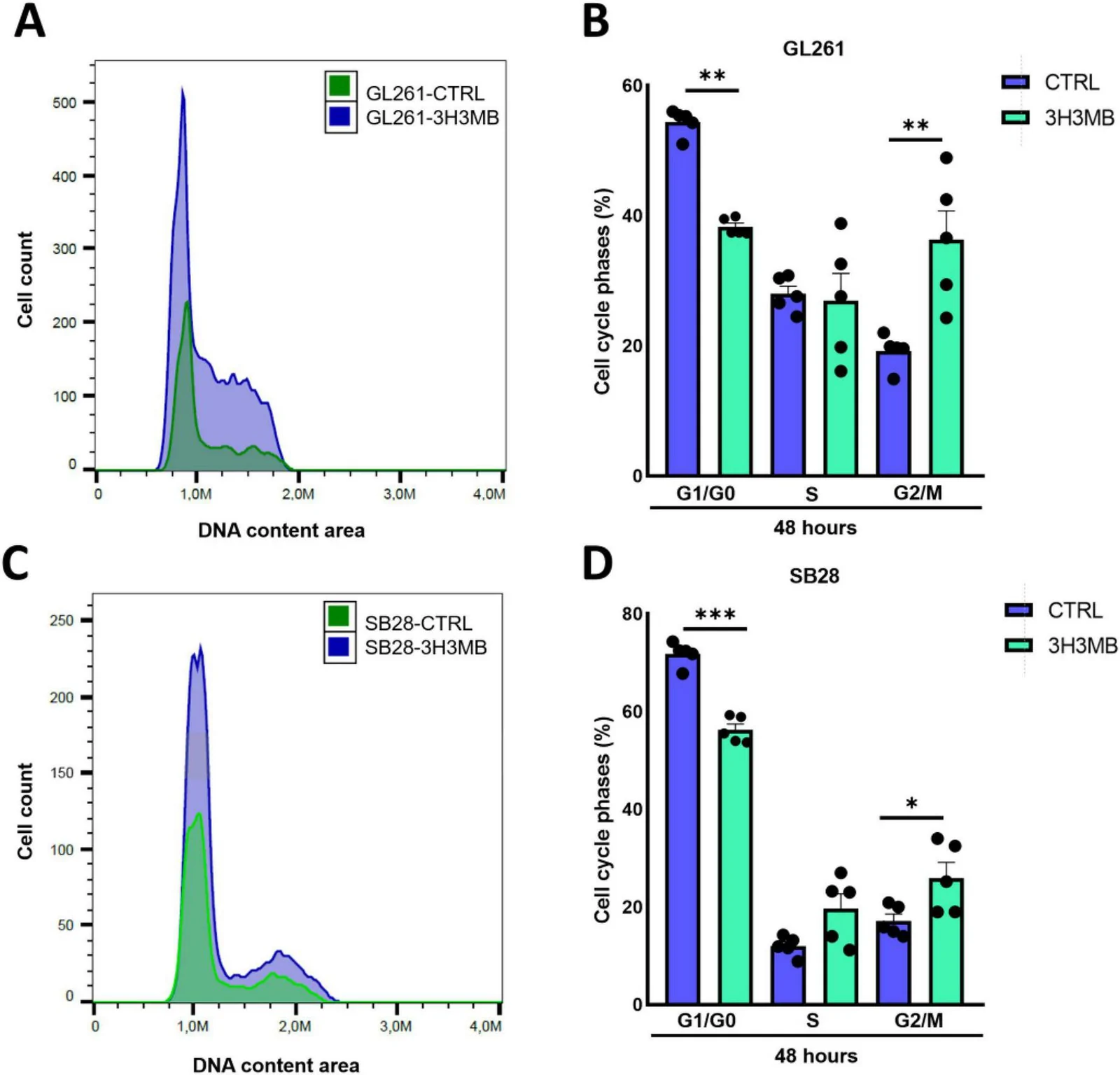
3H3MB treatment cell cycle distribution in mouse glioma cells. Representative DNA content histograms **(A,C)** and quantitative analysis of cell-cycle distribution **(B,D)** of GL261 and SB-28 glioma cells treated with 3H3MB. Cells were exposed to 3H3MB (250 μM) for 48 h and stained with propidium iodide (PI) for flow cytometric analysis. Quantification of the percentage of cells in the G0/G1, S, and G2/M phases is shown as mean ± SEM from *n* = 2 independent experiments, *n* = 5 samples. Statistical analysis was performed using one-way ANOVA followed by Tukey’s multiple comparisons test. *P < 0.05, **P < 0.01, ***P < 0.001 vs. respective control phase.

Given the close relationship between cellular proliferation and energy metabolism, mitochondrial respiration and glycolytic activity were subsequently evaluated using Seahorse extracellular flux analysis. In GL261 cells, treatment with 3H3MB significantly modified both oxygen consumption rate (OCR) and extracellular acidification rate (ECAR), indicating a metabolic adaptation involving both oxidative phosphorylation and glycolytic activity (Supplementary Figures 3A, B). Conversely, SB-28 cells displayed only minor, non-significant changes in OCR and ECAR profiles following treatment (Supplementary Figures 3C, D), suggesting that the metabolic response to 3H3MB is cell-line dependent despite the comparable antiproliferative effect observed in both models.

Finally, Annexin V/PI staining revealed no significant increase in either early or late apoptotic cells following 3H3MB treatment (Supplementary Figure 3), indicating that apoptosis is unlikely to represent the primary mechanism responsible for the reduced cell number under these experimental conditions. Overall, these findings suggest that 3H3MB exerts mainly cytostatic effects, characterized by altered cell-cycle progression and, in GL261 cells, by concomitant metabolic remodeling.

### 3.5 3 H3MB suppresses glioma cell migration and invasion in both mouse and human glioma cells

To assess the anti-migratory effect of 3H3MB, wound healing assays were performed in mouse (GL261, SB-28) and human (U-87 MG, GL-15) cell lines. To eliminate the confounding effects of proliferation, 5 mM hydroxyurea was added to all conditions. 3H3MB treatment impaired wound closure at both 24 and 48 h across all models compared to vehicle-treated controls (***p < 0.0001, one-way ANOVA followed by Tukey’s *post hoc* test), as shown in Figures 6A–E.

**FIGURE 6:**
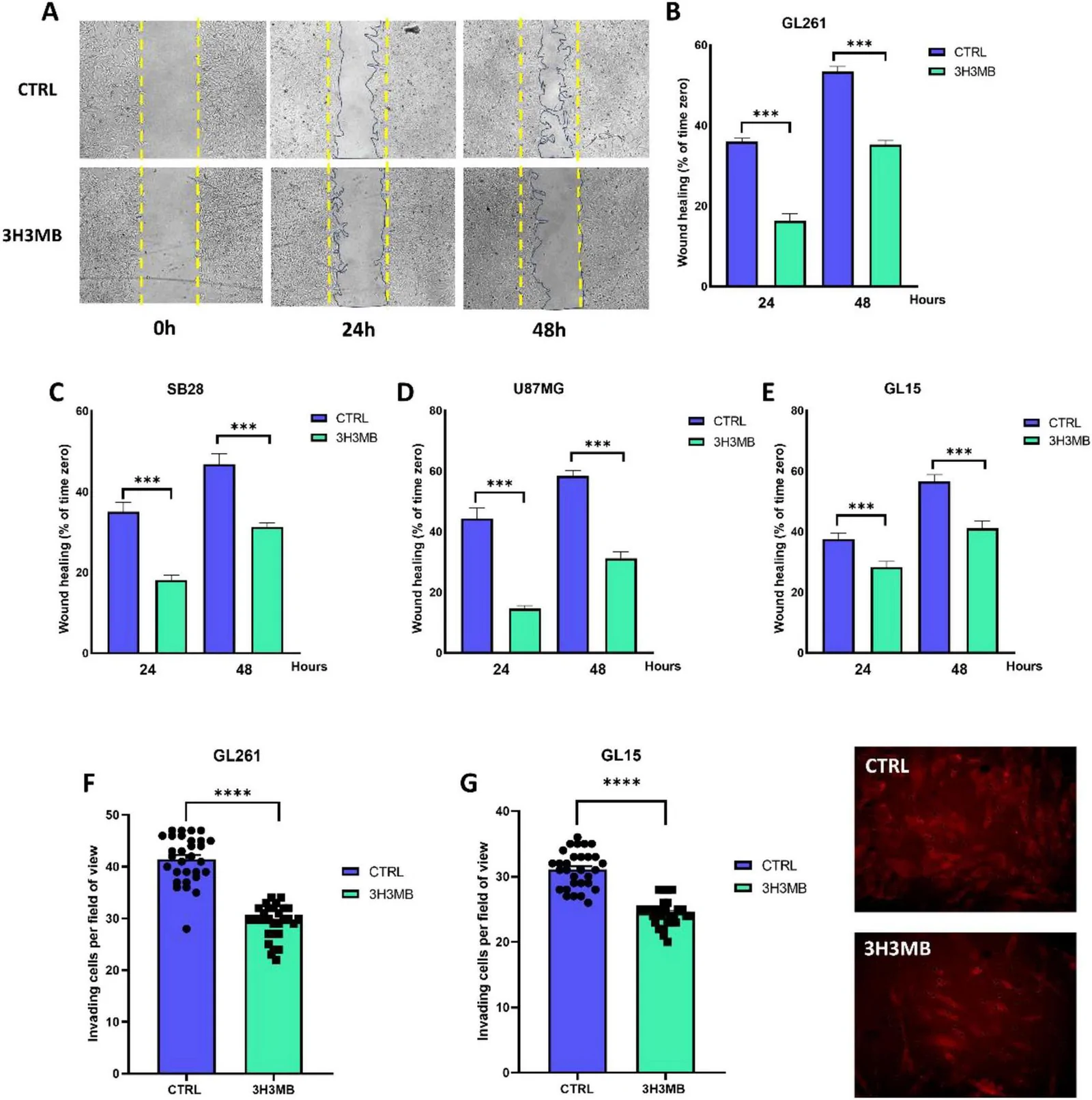
3H3MB inhibits glioma cell migration in mouse and human glioblastoma cell lines. **(A–E)** GL261 and SB-28 mouse glioma cells, along with GL-15 and U-87 MG human glioblastoma cell lines, were treated with. 3H3MB 500 μM and tested for basal migration by wound healing assay at 24- and 48-h post-treatment. Data are expressed as the mean percentage of wound-covered area relative to the 0-h time point. Error bars indicate mean ± SEM (***p < 0.0001 vs. respective CTRL). Representative phase-contrast images (20 × magnification) of GL261 were captured at each time point from four independent experiments. Bar graphs are generated by pooling the data from 4 independent experiments. **(F)** GL261 mouse glioma and **(G)** GL15 human glioblastoma quantification of cell invasion upon 3H3MB treatment (500 μM). Invasion was quantified as the mean number of invading cells per field of view. Data are shown as mean ± SEM ****p < 0.0001, with *n* = 3 independent experiments and at least 10 fields of view analyzed per condition per experiment. (Right) Representative image of GL-15-RFP invaded in control (upper) and in 3H3MB (lower) condition (20 × magnification).

To further investigate the impact of 3H3MB on glioma cell invasiveness, we conducted a Matrigel-coated transwell invasion assay using RFP expressing glioma cells. The results showed that 3H3MB treatment (500 μM) blocked FBS-induced tumor cell invasion in both mouse (GL261, CTRL = 41.47 ± 0.83 cells; 3H3MB = 29.40 ± 0.58 cells, ***p < 0.0001 by unpaired *t*-test, Figure 6F) and human cells (GL-15, Ctrl = 31.07 ± 0.51 cells; 3H3MB = 24.44 ± 0.39 ***p < 0.0001 by unpaired *t*-test, Figure 6G). Representative images are shown for GL-15-RFP cells in Figure 6G.

The consistency of these effects across species underscores the robust capacity of 3H3MB to inhibit glioma basal cell migration and FBS-induced invasion, highlighting its potential as a therapeutic agent.

## 4 Discussion

This study identifies the putative gut-associated metabolite 3-hydroxy-3-methylbutyrate (3H3MB) as a suppressor of glioma growth and progression, providing evidence that gut-brain communication influences CNS tumors. Through integrated metabolomic, cellular, and *in vivo* analyses, we demonstrated that brain glioma is associated with the reduction in fecal 3H3MB levels and that administration of this metabolite attenuates tumor proliferation, extending median survival time in glioma-bearing mice, being effective against both early tumor establishment and already established glioma.

The gut microbiota emerged as a crucial modulator of systemic metabolism, neuroinflammation and immune regulation, exerting profound influence on tumor initiation and progression across multiple organ systems (Derosa et al., 2022; Daisley et al., 2020; Yu and Schwabe, 2017). Several studies uncovered bidirectional communication in the gut–brain axis, linking intestinal microbe composition to brain physiology and pathologies (Marrocco et al., 2024, 2025; D’Alessandro et al., 2020; Ishaq et al., 2024; Dono et al., 2022). Microbiota-derived metabolites, such as short-chain fatty acids, tryptophan catabolites and amino acid derivatives, regulate the blood–brain barrier integrity, microglial activity, and immune cell function within the CNS (Marrocco et al., 2022; Braniste et al., 2014; Erny et al., 2015; Wu et al., 2020). Our finding that fecal 3H3MB levels are reduced in glioma-bearing mice adds a new dimension to this interaction, suggesting that the presence of a tumor within the brain could alter the composition of gut-derived metabolites, possibly through changes in microbial community composition (Dono et al., 2020; Aljarrah et al., 2024; Marrocco et al., 2026), tumor-driven metabolic reprogramming or alterations of the homeostatic host-microbiota cross-talk.

The detection of 3-hydroxy-3-methylbutyrate (3H3MB) in feces is most consistent with a microbial origin. In mammals, 3H3MB is generated in the liver as a minor product of leucine catabolism (∼5%–10%) and is rapidly released into circulation and excreted in urine, with negligible intestinal secretion (Nissen and Abumrad, 1997; Fuller et al., 2011). In contrast, gut microbiota actively metabolize leucine via fermentative pathways leading to branched-chain fatty acids such as isovalerate, and can produce 3H3MB or related intermediates locally. Notably, species such as *Ruminococcus gnavus* have been implicated in the generation of 3H3MB-like metabolites (Smith and Macfarlane, 1997; Ding et al., 2026). 3H3MB has been extensively characterized for its anabolic and cytoprotective roles in skeletal muscle, where it promotes protein synthesis, mitochondrial biogenesis, and membrane integrity through activation of mTOR and MAPK pathways (Holeček, 2017; Prado et al., 2022). In tumors, 3H3MB has inhibitory effects on cell proliferation and growth. Recent work demonstrated that 3H3MB supplementation suppressed pancreatic ductal adenocarcinoma progression and enhanced anti-tumor immunity in obesity-associated cancer models (Coleman et al., 2021). Consistent with these observations, our study reveals that 3H3MB inhibits glioma cell proliferation in tumor mass, reduces cell viability, migration, and invasion across both mouse and human cells, suggesting a common activity. Importantly, our *in vitro* experiments further support a direct effect of 3H3MB on glioma cell growth. The reduction in cell viability was observed across all tumor cell lines tested, indicating that the anti-proliferative activity of 3H3MB is not restricted to a single experimental model. Conversely, human astrocytes were not significantly affected under the same experimental conditions, suggesting a degree of preferential sensitivity of glioma cells to 3H3MB. While the specific molecular targets remain to be elucidated in glioma, several plausible pathways can be envisioned. Previous studies in skeletal muscle models have shown that 3H3MB can influence mitochondrial function and biogenesis, including in C2C12 myotubes through pathways involving PPARβ/δ and CDK4 (Zhong et al., 2019), while studies in rats have linked 3H3MB to activation of the mTOR pathway and stimulation of muscle protein synthesis (Pimentel et al., 2011). Similarly, 3H3MB has been reported to promote skeletal muscle regeneration through the IGF-Akt pathway (Yamada et al., 2022). In cancer models, 3H3MB supplementation has been shown to reduce tumor growth and tumor-cell proliferation in Walker-256 tumor-bearing rats, an effect associated with modulation of NF-κB signaling (Nunes et al., 2008). Clinical studies in patients with cancer have mainly investigated 3H3MB in the context of preservation of muscle mass and function rather than direct tumor-cell effects (Prado et al., 2022). Our Seahorse analyses extend these observations to glioma cells by showing that 3H3MB significantly alters both OCR and ECAR in GL261 cells, suggesting that 3H3MB can influence tumor-cell bioenergetics. Interestingly, these changes were not significant in SB-28 cells, despite the anti-proliferative effect observed in both models. This finding suggests that metabolic remodeling is cell-line dependent and may represent an adaptive response rather than the unique mechanism responsible for the anti-proliferative activity of 3H3MB. The marked reduction in Ki67-positive cells observed in 3H3MB-treated glioma cells supports the hypothesis that this metabolite is associated with reduced proliferation. Consistent with this observation, our cell-cycle analysis showed that 3H3MB treatment altered cell-cycle distribution in both GL261 and SB-28 cells, with a redistribution of cells across the G0/G1, S, and G2/M phases. These findings suggest that the reduction in glioma cell growth is associated with impaired cell-cycle progression. Notably, Annexin V/PI analysis did not reveal a significant increase in early or late apoptotic cells, suggesting that the anti-proliferative effect of 3H3MB is predominantly cytostatic rather than driven by apoptosis under the experimental conditions tested (Supplementary Figure 4). Furthermore, given the known impact of microbial metabolites on immune modulation (Erny et al., 2015, 2017), 3H3MB might influence glioma-associated macrophages, microglial polarization, or T-cell infiltration (Gimple et al., 2019; Trejo-Solis et al., 2023). This possibility is particularly intriguing in light of recent evidence demonstrating that specific microbial populations regulate glioma progression by shaping antitumor immunity. Intestinal *Bacteroides* species were shown to drive glioma progression through modulation of CD8^+^ T-cell infiltration, while distinct human microbiota configurations alter immune-cell composition and transcriptional programs within the glioma microenvironment (Chatterjee et al., 2025; Green et al., 2025). Moreover, gut microbial composition influences the response to anti-PD-1 immunotherapy in glioma-bearing mice, highlighting the strong interaction between microbial signals and antitumor immune responses (Dees et al., 2021). Therefore, beyond its direct effects on glioma cells, the antitumor activity of 3H3MB may involve modulation of immune pathways that remain to be elucidated. Taken together, these findings suggest that 3H3MB primarily restrains glioma cell proliferation through alterations in cell-cycle progression, with no contribution of apoptosis. The differential metabolic response observed between GL261 and SB-28 cells further indicates that metabolic adaptation is not uniformly required for the anti-proliferative effect of 3H3MB. This cell-line-dependent metabolic response may reflect the well-established metabolic heterogeneity of glioblastoma (Garofano et al. 2021) and highlights the possibility that distinct tumor cell populations may respond to 3H3MB through partially overlapping mechanisms.

From a translational perspective, our findings carry putative clinical implications. 3H3MB is an endogenous compound, already used as a dietary supplement in humans, with a well-documented clinical safety profile in the context of muscle wasting, cachexia, and metabolic disorders (Berk et al., 2008; Pascoe et al., 2021; Prado et al., 2022). The demonstration that oral administration of 3H3MB significantly reduces tumor volume and prolongs median survival in immunocompetent mice suggests that interventions targeting microbiota-derived pathways could serve as a feasible and non-toxic adjuvant therapy for GBM. Considering oral bioavailability, the capability to reach systemic circulation and cross the blood–brain barrier (Barranco et al., 2022; Higuchi et al., 2021), repurposing 3H3MB for neuro-oncological applications represents a realistic translational opportunity. However, it will be essential to investigate whether 3H3MB crosses the blood–brain barrier at therapeutic concentrations and to evaluate its potential synergy with current standard-of-care therapies such as temozolomide, radiotherapy, and immune checkpoint inhibitors (Sadowski et al., 2024; Tan et al., 2020). This aspect is particularly relevant because growing evidence indicates a bidirectional interaction between temozolomide treatment and the gut microbiota. Glioma itself and temozolomide administration induce significant shifts in microbial composition, whereas microbiota-dependent immune mechanisms can influence the therapeutic efficacy of temozolomide (Patrizz et al., 2020; Li et al., 2021; Hou et al., 2023). Therefore, future studies should investigate whether 3H3MB supplementation enhances standard therapies through microbiota-dependent mechanisms. Moreover, modulation of the gut microbiome composition to enhance endogenous 3H3MB production could represent an innovative therapeutic approach in patients at high risk of glioma recurrence.

Nonetheless, several limitations must be acknowledged. The microbial origin of 3H3MB remains to be experimentally demonstrated. Although our data clearly demonstrate the anti-tumor efficacy of 3H3MB, the underlying molecular mechanisms remain incompletely defined. Whether the observed reduction of endogenous 3H3MB contributes causally to glioma initiation or merely reflects tumor-driven metabolic remodeling remains an open question. Since the *in vivo* studies were conducted in immunocompetent mice, immune-mediated effects may contribute to the anti-tumor activity of 3H3MB; future studies should define the role of immune cells in its therapeutic efficacy. Furthermore, while mouse models recapitulate several hallmarks of human GBM, interspecies differences necessitate validation of these findings in patient-derived xenografts and organoid models (Frederico et al., 2023).

In conclusion, our data reveal that 3H3MB, a gut microbiota–associated and host leucine metabolite, exerts robust anti-tumor effects in glioma, suppressing proliferation, migration, and invasion, reducing tumor volume, and extending median survival *in vivo*. These findings expand our understanding of the gut–brain interplay in glioma and identify 3H3MB as a promising therapeutic candidate. By bridging microbiome science and neuro-oncology, this work provides a foundation for the development of microbiota-targeted and metabolism-based strategies aimed at improving outcomes for GBM patients.

## Data Availability

The original contributions presented in this study are included in this article/Supplementary material, further inquiries can be directed to the corresponding authors.
