# Supplementary material for: 3-hydroxy-3-methylbutyrate (3H3MB) suppresses glioma growth and extends survival in mice
Source: Front Mol Neurosci. 2026 Sep 17;19:1917452. doi: 10.3389/fnmol.2026.1917452 (PMC13627411; doi:10.3389/fnmol.2026.1917452)
Supplement: Supplementary file 1 [file Data_Sheet_1.pdf]

3-hydroxy-3-methylbutyrate (3H3MB) suppresses glioma growth and extends survival in mice

Rizwan Khan[1,§], Xingzi Lin[1,§], Francesco Marrocco[1,] Ottavia Giampaoli[2,3], Javeria Maqbool[1], Alice Reccagni[4], Fabio Sciubba[2,3], Adriano Patriarca[3,5], Michele De Rosa[3,5], Alfredo Miccheli[2,3],Gabriele Favaretto6, Fabrizio Antonangeli6, Cristina Limatola[7,4§*], Giuseppina D'Alessandro[7,4§*].

[1] Department of Physiology and Pharmacology, Sapienza University, Rome, Italy

[2] Department of Environmental Biology, Sapienza University, Rome, Italy
[3] NMR-based Metabolomics Laboratory, Sapienza University, Rome, Italy
4 Neuromed, Pozzilli, IS, Italy
[5] Department of Chemistry, Sapienza University, Rome, Italy
6 CNR

[7] Department of Physiology and Pharmacology, Sapienza University, laboratory affiliated to Istituto Pasteur Italia, Rome, Italy.

Supplementary Figures and Figure Legends

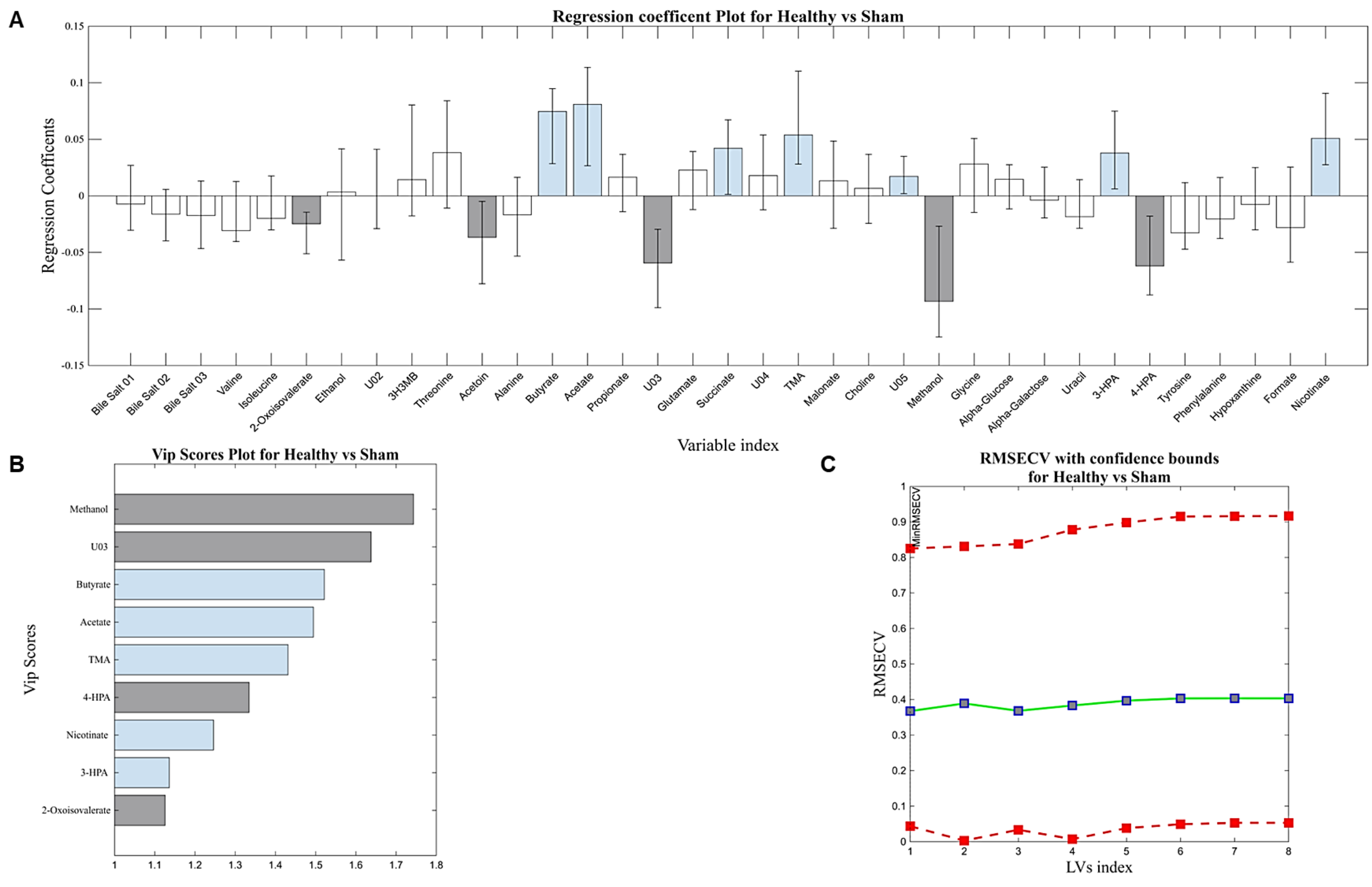

Supplementary figure 1: PLS-DA panel for Healthy vs Sham mice model for ^{1}H NMR metabolomics. Validation results during repeated double cross validation procedures yielded a model for the 1st LV with accuracy: 0.84 ± 0.16, precision:

1.00 ± 0.23, sensitivity: 1.00 ± 0.24, specificity: 1.00 ± 0.26, AUC: 1.00 ± 0.16. Plot A is the regression coefficient plot, showing in azure metabolites with consistently higher coefficients for Sham mice, in grey metabolites with consistently higher coefficients for healthy mice and in white metabolites with inconsistent sign during cross validation procedures. Plot B is the Vip scores plot, showing metabolites ranked by Importance in Projection, with the same color code as plot A. Plot C is the Root Mean Square Error during Cross Validation procedures (RMSECV), plotting the mean and confidence interval values for the repeated CV iterations.

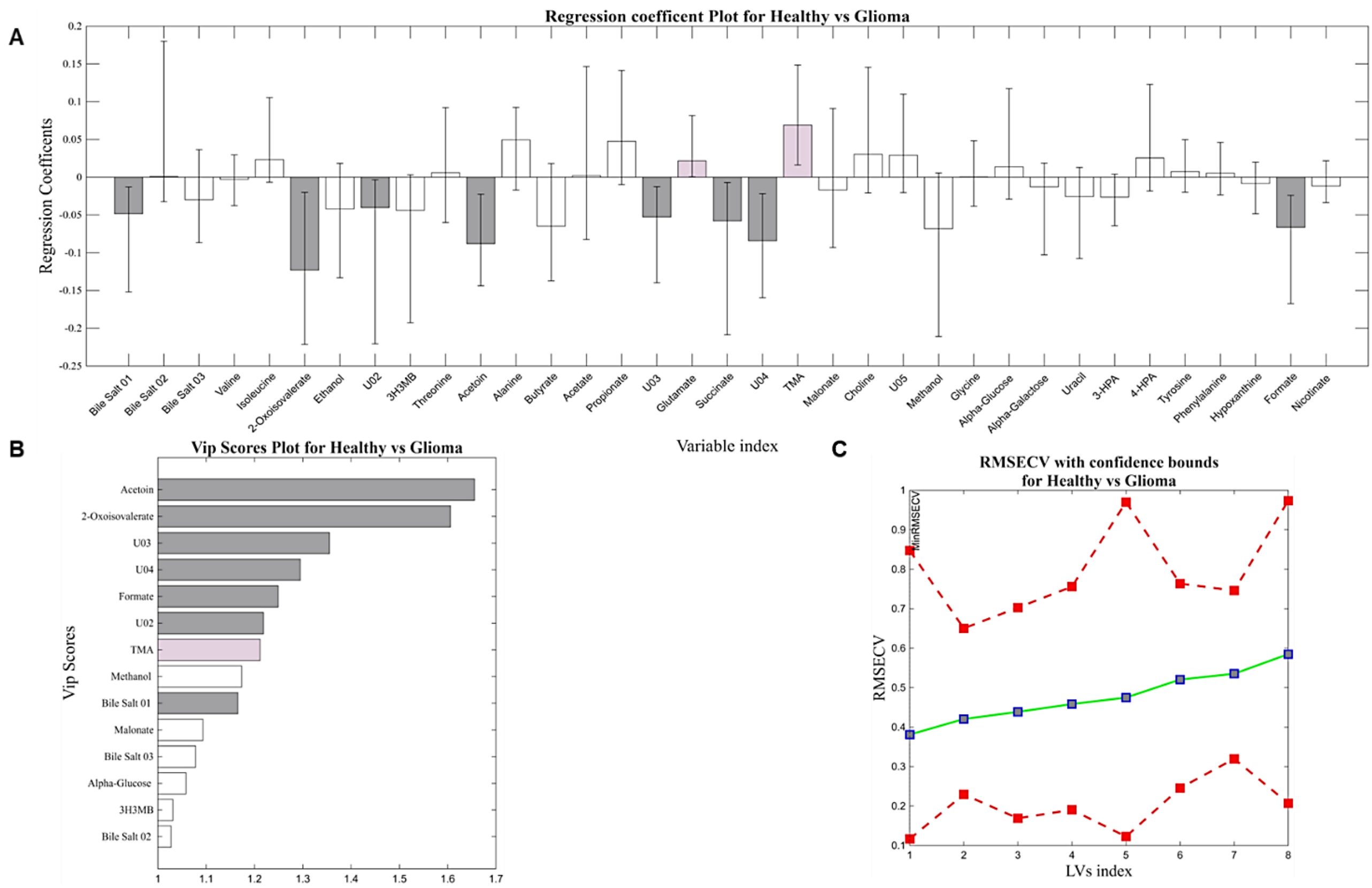

Supplementary figure 2: PLS-DA panel for ^{1}H NMR metabolomics. Validation results during repeated double cross validation procedures yielded a model for the 1st LV with accuracy: 0.77 ± 0.12, precision: 0.75 ± 0.16, sensitivity: 1.00 ± 0.16, specificity: 0.67± 0.22, AUC: 0.90 ± 0.13. Plot A is the regression coefficient plot, showing in lilac metabolites with consistently higher coefficients for Glioma mice, in grey metabolites with consistently higher coefficients for healthy mice and in white metabolites with inconsistent sign during cross validation procedures. Plot B is the Vip scores plot, showing metabolites ranked by Importance in Projection, with the same color code as plot A. Plot C is the Root Mean Square Error during Cross Validation procedures (RMSECV), plotting the mean and confidence interval values for the repeated CV iterations.

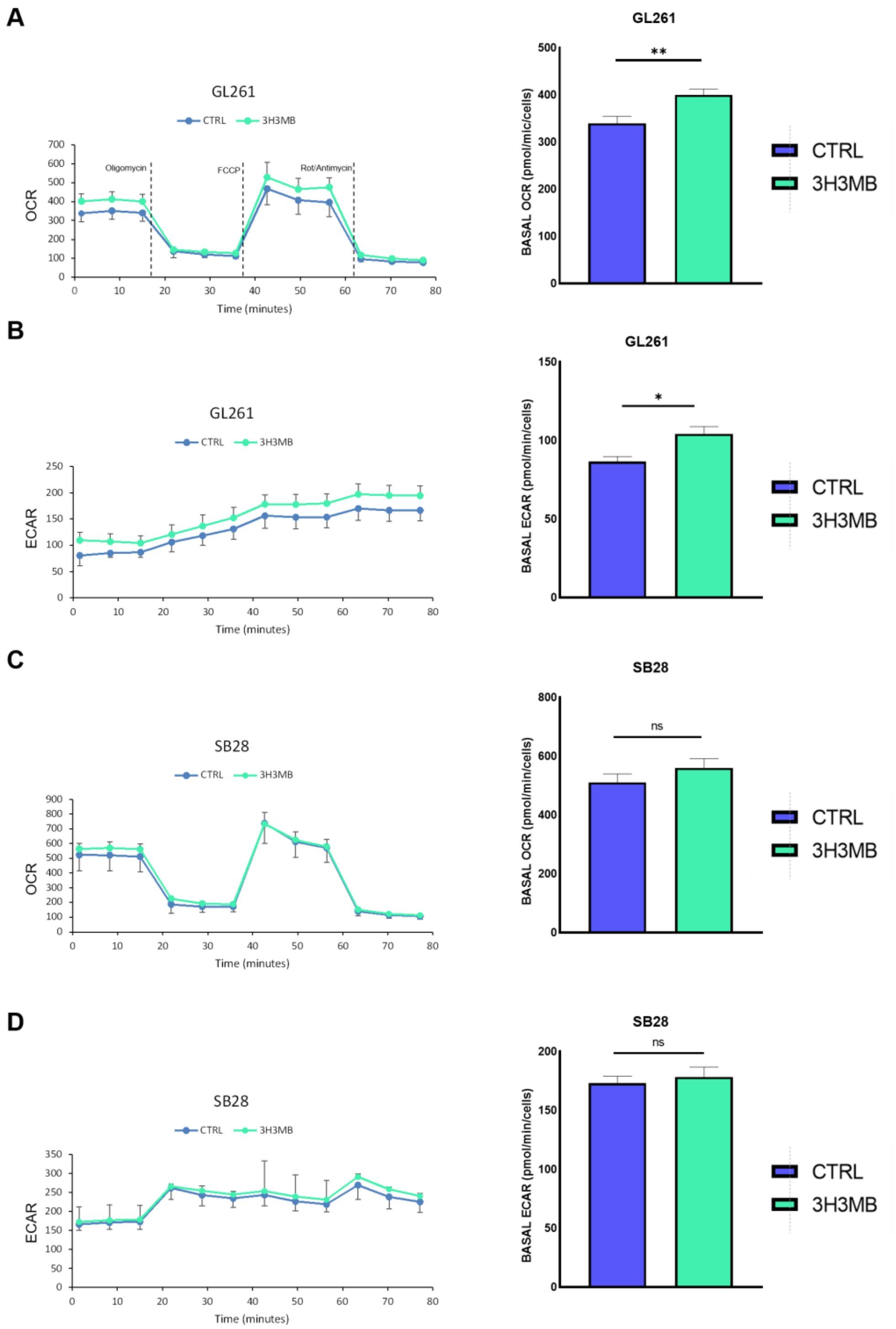

Supplementary figure 3: Representative Seahorse extracellular flux analysis profiles (left panels) and quantitative analysis (right panels) of oxygen consumption rate (OCR) and extracellular acidification rate (ECAR) in GL261 and SB28 glioma cells treated with 250 µM 3H3MB for 36 h. (A) OCR measurements and basal respiratory capacity quantification in GL261 cells. (B) ECAR measurements and glycolytic activity quantification in GL261 cells. (C) OCR measurements and basal respiratory capacity quantification in SB28 cells. (D) ECAR measurements and glycolytic activity quantification in SB28 cells. During the mitochondrial stress test, oligomycin, FCCP, and rotenone/antimycin A were sequentially injected at the indicated time points. Cells were treated with 3H3MB and analyzed using the Seahorse XF Extracellular Flux Analyzer. Data are presented as mean ± SEM. Statistical analysis was performed using an unpaired Student's *t*-test. $P < 0.05$; $P < 0.01$ versus the respective control (CTRL) group.

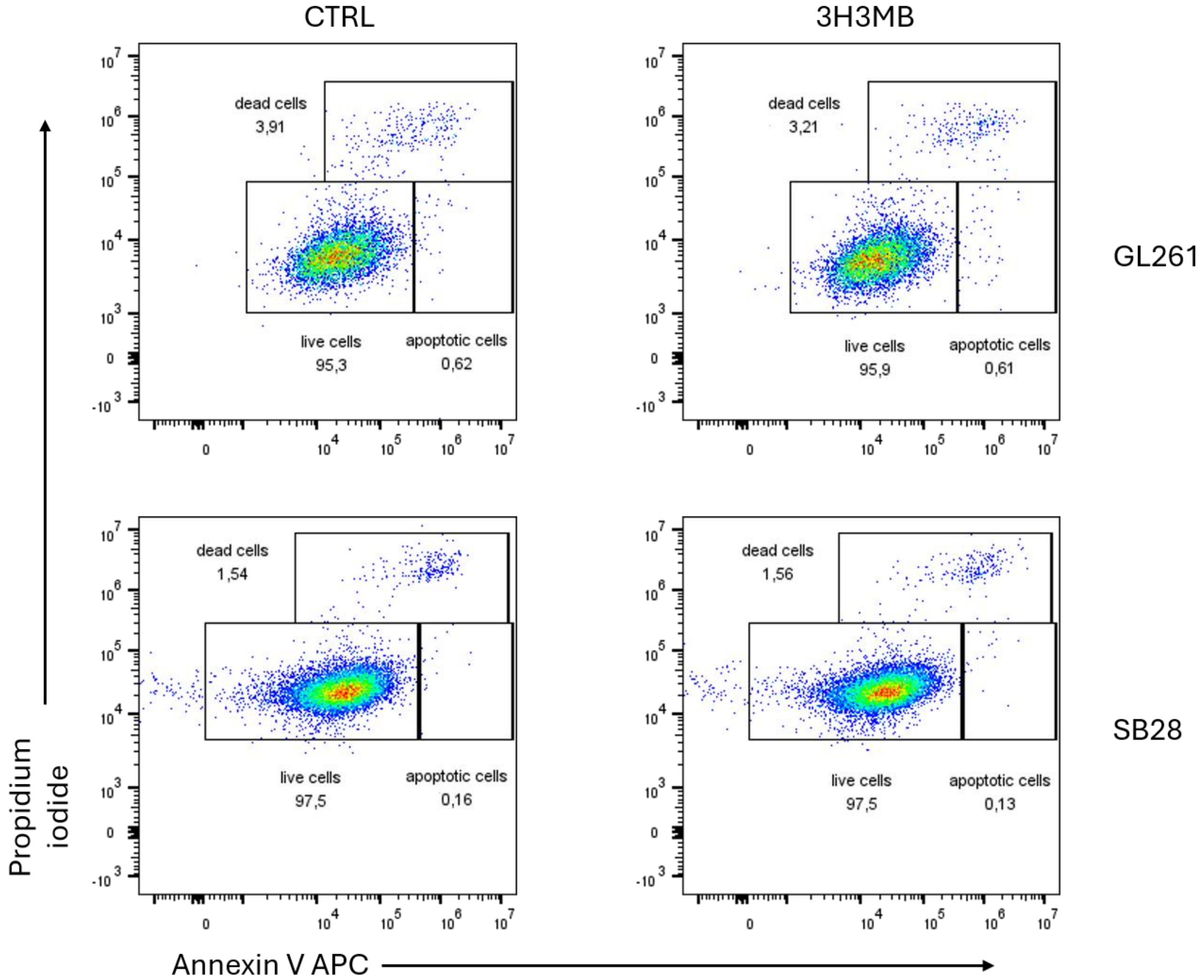

Supplementary figure 4: Apoptotic and dead cells were analyzed by flow cytometry using the APC Annexin V Apoptosis Detection Kit with PI (BioLegend, Cat. No. 640932), following the manufacturer's protocol. GL261 and SB28 cells were treated with 250 µM 3H3MB or vehicle for 48 h before analysis. No differences were detected between the treated and control groups in either glioma cell line.

Supplementary Methods

Apoptosis detection

For the detection of apoptosis, cells were harvested, washed with Binding Buffer (10 mM HEPES, 140 mM NaCl, and 2.5 mM CaCl2), and apoptotic and dead cells were analyzed by flow cytometry using the APC Annexin V Apoptosis Detection Kit with PI (BioLegend, Cat. No. 640932), following the manufacturer's protocol.

Seahorse XF Analyzer Respiratory Assay

Cellular oxygen consumption rate (OCR) and extracellular acidification rate (ECAR) were detected using XF Cell Mito Stress Test (Agilent) measured by the extracellular flux analyzer XFePro (Seahorse Bioscience, Houston, TX, USA) in the HypACB facility at Sapienza University. GL261 and SB28 cells were XFe culture 96-wells miniplates (17000/well), and when adherent, treated with 250 µM 3H3MB for 36 hours. The sensor cartridge for XFe analyzer was hydrated in a 37 °C non-CO2 incubator a day before the experiment. According to the manufacturer instructions, stressors concentrations were optimized and added as follows: 1 µM oligomycin as complex V inhibitor, 0.5 or 1 µM FCCP as uncoupler agent (for S or G cells, respectively) and 0.5 µM rotenone/antimycin A (inhibitors of complex I and III). During sensor calibration, cells were incubated in a 37 °C non-CO2incubator in 180 µl assay medium (XF base medium supplemented with 10 mM glucose, 10 mM pyruvate and 2 mM L-glutamine at pH 7.4, was used to wash the cells and replace the growth medium). Each sample/treatment was analysed in 10 wells for experiment and refers to its own untreated control; the figure represents the mean of 10 replicates ±SD. Statistical analysis has been performed on each experiment by using one way ANOVA followed by Bonferroni post-hoc comparison test.
